# The Impact of Alcohol Hangover on Simulated Driving Performance during a ‘Commute to Work’—Zero and Residual Alcohol Effects Compared

**DOI:** 10.3390/jcm9051435

**Published:** 2020-05-12

**Authors:** Chris Alford, Callum Broom, Harriet Carver, Sean J. Johnson, Sam Lands, Rebecca Reece, Joris C. Verster

**Affiliations:** 1 HAS - Health and Social Sciences, University of the West of England, Bristol BS16 1QY, UK; Callum_Broom@hotmail.com (C.B.); Hatty_Carver@hotmail.com (H.C.); Sam.Lands@alliancepharma.co.uk (S.L.); Rebecca.Reece@uwe.ac.uk (R.R.); 2 Centre for Trials Research, Cardiff University, Cardiff CF14 4YS, UK; JohnsonS11@cardiff.ac.uk; 3 Division of Pharmacology, Utrecht Institute for Pharmaceutical Sciences (UIPS), Utrecht University, 3584CG Utrecht, The Netherlands; j.c.verster@uu.nl; 4 Centre for Human Psychopharmacology, Swinburne University, Melbourne, VIC 3122, Australia

**Keywords:** alcohol, awareness of impairment, hangover, driving, residual alcohol

## Abstract

Driving is increasing across the world and road traffic accidents are a major cause of serious injuries and fatalities. The link between alcohol consumption and impaired driving has long been established and has led to legislation in many countries, with enforcement of legal limits based on blood alcohol concentration levels. Alcohol hangover research is an emerging field with a range of laboratory and naturalistic studies now clearly demonstrating the significant impairments that can result from hangover, even when alcohol levels are measured at or close to zero the day following a social drinking occasion. Driving is a commonplace activity but requires competency with a range of complex and potentially demanding tasks. Driving impaired can have serious consequences, including death and serious injury. There have been only limited alcohol hangover driving studies. The studies presented examined the consequences of alcohol hangover with a driving simulator contrasting a group with zero residual alcohol (N = 26) next day and another with residual alcohol (N = 26) assessed with breathalyzer in the morning before undertaking a 20 min commute to work. All participants completed a morning drive after a night without alcohol consumption and another after a night of social drinking. The driving scenarios were relatively demanding including traffic and pedestrians, traffic lights and other potential hazards in a mixed rural and urban journey. Subjective hangover and workload were assessed in addition to a range of driving performance variables, including divided attention, steering control and driving violations. Analyses contrasted driving in the no alcohol condition with the residual alcohol condition. The combined groups data (N = 52) was contrasted with the zero and residual alcohol groups. Significant contrasts were found for a range of driving measures, including divided attention, vehicle control, and driving violations as well as perceived workload. The pattern of impairment was broadly similar across both groups, indicating that whether or not residual alcohol was present, consistent driving impairment was seen. The relatively high number of significant variables may reflect the increased cognitive demand of the 20 min commute drive including busy and complex urban environments. This was also reflected in the significant increase in perceived workload recorded across the 6 dimensions of the National Aeronautics and Space Administration Task Load Index (NASA-TLX). Associations between subjective measures and driving performance with hangover suggested a potential lack of awareness of impairment, though were limited in number. The overall findings indicate that the levels of impairment seen reflect those seen with alcohol impaired driving, even when breath alcohol is zero.

## 1. Introduction

Road traffic accidents are a leading cause of mortality, with around 1.35 million deaths recorded worldwide in 2016, ranked in the top 10 causes of mortality, the leading cause of death for 5–29 year olds, and with up to 35% of road accident deaths estimated as being alcohol related [1]. Whilst there has been much research into the impairing effects of alcohol intoxication on driving performance [2,3,4,5], there has been only limited research into the effects of alcohol hangover on driving [6,7,8,9,10,11]. Alcohol hangover is the most commonly experienced consequence of alcohol consumption [12] and is defined as the combination of negative mental and physical symptoms which can be experienced after a single episode of alcohol consumption, starting when blood alcohol concentration (BAC) approaches zero [13,14]. The annual costs of alcohol hangover to the economy in terms of absenteeism and presenteeism have been estimated at 169 billion dollars for the US and 4 billion pounds for the UK [15,16], corresponding with a significant loss of productivity [17]. The neurocognitive and psychomotor effects of alcohol hangover have been demonstrated in both laboratory and field studies [18,19], showing significant levels of impaired function on a range of tasks and impacting mood and subjective state in some but not all studies.

The one hour (100 km) on-road highway driving test is considered the ‘gold standard’ of driving assessments and has been the basis of a large body of research in the Netherlands examining the impact of acute dosing as well as residual effects of a range of psychoactive compounds including alcohol [20]. The test has also been successfully implemented in driving research demonstrating the acute and after effects of alcohol [9,21]. The principal measure is the standard deviation of lateral position (SDLP) that captures the weaving behavior of the car as it travels along the highway or motorway environment. The more impaired a driver is then the greater the weaving and increased measure of SDLP. The highway driving test was developed to measure monotonous driving performance with low external stimuli, in order to capture basic driving performance. However, other driving situations such as city driving comprise more skills than primarily steering control and vigilance performance. Different driving environments impose different levels of cognitive demand on the driver. The one hour driving test is undertaken in a relatively undemanding driving environment for a competent driver, such as a highway or motorway setting with an identified and clearly marked driving lane and without oncoming traffic. Driving assessments made under more challenging conditions have found significant impairment in driving control at blood alcohol concentration as low as 0.02%–0.03% BAC [3,22], and it has been shown that driving distractions can produce a two-fold increase in alcohol induced driving impairment [4].

A wide range of cognitive and psychomotor functions including planning, working memory, attention and perceptual motor tasks become more important in more cognitively demanding tasks such as a ‘commute to work’. Modern simulators include a range of measures which complement SDLP and may provide a more comprehensive assessment of driving abilities needed in these circumstances. Whilst the one hour test has a significant bibliography to support it and provides comparators for assessing any new psychoactive compounds and medications, this does not represent the typical morning ‘commute to work’, which can include a range of rural as well as busy urban environments and is of shorter duration [23]. There is a need to assess shorter driving durations as the UK 2018 National Travel Survey [24] found that the average car journey was found to be just over 20 min. Typical commutes to work have been reported as averaging around 20–30 min in the UK in 2018 [23]. Given that a significant number of the working population will use a car for their morning commute, then the impact of alcohol hangover on a typical commute to work requires further investigation.

To date, there have been relatively few hangover driving studies [6,7,8,9,10,11]. Verster et al. [10] surveyed 343 Dutch truck drivers who reported impaired driving after drinking the previous night. Of concern, the drivers that participated in this study acknowledged continued driving, both professional and private, despite being aware that being hungover impaired their driving. Early driving studies reported impairment during hangover when performing swerve maneuvers around cones on a closed road, and performance on a high speed driving test in a simple driving simulator [7,8]. More recently, Verster et al. [9] reported significant driving impairment (increased SDLP) on a standardized 100-km highway driving test in the STISIM driving simulator. Significant increases in SDLP occurred mostly in the last half of the simulated drive. Robbins et al. [11] examined driving during hangover employing a 15 min test of simulated motorway driving, including hazards. They found that in the hangover condition participants, who mostly had residual alcohol though below the legal limit, drove at higher speeds and for longer periods over the speed limit, with greater speed variation interpreted as increased likelihood of traffic violations, although attention was not impaired. Together, these studies suggest that driving ability during hangover is impaired at various performance levels, which may also be present during a ‘commute to work’ drive.

Awareness of impairment plays an important role in determining if someone drives as well as affecting their driving behavior. Self estimates of acute alcohol intoxication vary with the level of consumption such that lower levels below the UK driving limit 0.08% BAC may be accurate or overestimates, transitioning to underestimates for higher levels [25,26]). There has been relatively little published research into the effects of alcohol hangover on self estimates of induced impairment, and yet this is an important aspect given that alcohol hangover cannot easily be quantified in the same way as a breathalyzer might assess alcohol induced impairment.

The current study was set out to assess the next-day consequences of a social drinking session on driving simulator performance on a representative commute to work drive. The study employed the commonly accepted naturalistic design [27]. The primary research question was to examine whether ‘commute to work’ driving is impaired during alcohol hangover. Previous driving studies investigating both acute alcohol intake and alcohol hangover have found significant decrements in performance despite increased mental effort [9]. This is therefore an important measure, particularly where cognitive demand is increased, and was therefore included in the current study. Awareness of impairment is also an important driving safety related factor and so the association between subjective state and driving performance was assessed.

Finally, the current definition of alcohol hangover states that the alcohol hangover starts when BAC approaches zero [13,14]. The few currently published driving studies did not differentiate between subjects that have a BAC of zero and subjects with residual alcohol still present [9,11] and included all subjects in the statistical analysis where they were treated as a single sample. However, given the established driving impairments found with acute alcohol administration at low dosages [3,22], it may be important to distinguish residual alcohol effects from hangover effects. Therefore, the current study directly contrasted hungover subjects with residual BAC with those with a BAC of zero.

## 2. Methods

### 2.1. Design and Participants

A within-subjects naturalistic design was used to investigate driving performance and subjective state contrasting two conditions: alcohol hangover (the morning after a social drinking occasion) and an alcohol-free control day (the morning after no alcohol consumption). Participants were free to arrange or postpone assessment days as they chose, depending on whether they had consumed alcohol on their social drinking occasion. The order of conditions was counterbalanced across participants, as was the order for the driving scenarios used to assess driving. The data represents the combination of three studies which were approved by the University of the West of England Faculty Ethics Committee (Approval numbers: ASP12-276, HAS.14.12.74, HAS.16.12.069). Informed consent was obtained from all participants, and the studies were undertaken in accordance with the British Psychological Society Code of Ethics and Conduct (2009). No financial payments were made for participation.

Participants included both students as well as others recruited through contacts of the experimenters. A total of N = 52 participants (21 men and 31 women) age range of 18–25 years, with a mean age of 20.9 were included. They were driving license holders with 1–6 years driving experience who self-reported as being in good health, not pregnant or using illicit drugs at the time of the investigation, non-smokers, with a daily consumption of less than 5 cups of coffee or other caffeinated drinks. Subjects were social drinkers and self-reported to experience hangovers regularly. There were 26 participants in each of the BAC zero and residual alcohol groups.

### 2.2. Procedure

Prospective participants completed screening questionnaires collecting demographic information and assessing health and pregnancy status, alcohol, caffeine, drug use and alcohol hangover experience. Screening also included a short 5 min simulated drive in the STISIM to check for simulator sickness propensity, promote adaptation, and familiarize participants with the driving simulator test [28,29].

During the study, participants attended the driving simulator twice in the morning for full driving assessment, once after an evening without alcohol consumption (no alcohol) and once after an alcohol consumption occasion (alcohol/hangover). They did not ride or drive to the university on post alcohol assessment days and their health status was checked verbally and by observation on attendance, followed by breathalyzer assessment. They were asked to follow their regular procedures on assessment days, although excluding caffeine consumption on the test days until after participation was completed. They completed subjective assessments rating hangover severity and subjective workload after completing the driving simulator test. Following completion of assessments participants health status was again checked before they were permitted to leave the University. On study completion they were provided with a debrief sheet including contacts for the experimenters, as well as advice on support for excessive alcohol use.

### 2.3. Breathalyzer Assessments

Breath alcohol concentration was measured with a Lion Alcometer 400/500 (Lion Laboratories, UK) by the experimenters who kept their hand over the visual display so that participants were blind to readings. Assessments were conducted directly before the driving simulator test.

### 2.4. Driving Simulator Test

**Figure 1:**
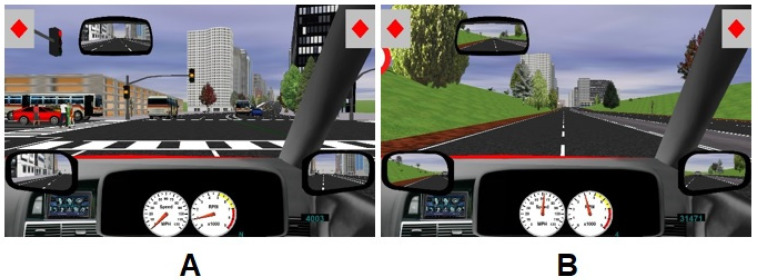
Examples from the STISIM driving test scenario. The STISIM driving test scenario included both city driving scenes (**A**) with a relatively high number of external stimuli (e.g., traffic lights, pedestrians crossing the road and rural roads (**B**) with less external stimuli (e.g., no traffic lights, lower traffic density). On the top left and right of the screen, symbols for the divided attention task are depicted to which subjects had to respond by pressing a button on the steering wheel.

A STISIM DriveTM driving fixed base simulator was used to measure driving performance (Systems Technology Inc, Hawthorne, CA, USA). The simulator comprises a driving frame housing the adjustable seat, standard foot controls (clutch, brake, accelerator), and the gear lever. The Driving scenario (see Figure 1) is displayed on a 40” LG monitor. The speed of the vehicle and current gear selection are displayed within the virtual dashboard at the bottom of the screen. The balanced scenarios comprised 60,000 feet of mixed rural and urban driving environments with speed limit set to 50 mph. This provided a driving assessment period of around 20 min in a virtual driving environment with UK signage and driving on the left hand carriageway. Potential hazards included crossings, stop signs, traffic lights, parked vehicles in the road, other vehicles as well as pedestrians and dogs crossing the road. A divided attention task was included with symbols displayed in the top left and right of the screen. The default symbol is a diamond and when either symbol changes to a triangle the participant is required to press the corresponding response button on the steering wheel. A range of buildings dominated the urban landscape, whilst trees and fields were prominent in the rural sections of the driving environment.

The STISIM driving simulator records a range of parameters, including variables related to divided attention (mean response time, and the number of correct, incorrect and missed responses), basic vehicle control (mean speed and deviation, mean lane position and standard deviation of lateral position (SDLP)), driving errors (percentage of center line crossings (offside), percentage off road (nearside), excursions from lane) and driving violations (number of accidents and collisions, speed limit exceedances and tickets (e.g., failure to stop at stop sign or jumping red lights)).

### 2.5. Subjective Assessments

The Alcohol Hangover Severity Scale (AHSS) with an 11 point scale of 0–10 for a variety of symptoms, including fatigue, nausea and thirst with a total of 12 descriptors was used to assess subjective hangover [30]. Fatigue was selected as a single dimension to provide a measure of fatigue and sleepiness which are important consequences of hangover and associated with driving impairment [31,32]. Participants were also required to recall their alcohol consumption on their social nights out with the aid of a retrospective diary. The National Aeronautics and Space Administration Task Load Index (NASA-TLX) was used to assess perceived workload with an 101 point scale 0–100 with 5 unit divisions, for 6 workload dimensions including effort and performance, which are both important for assessing subjective impairment as well as the overall average or Raw TLX [33,34].

### 2.6. Statistical Analyses

Statistical analyses were undertaken using IBM SPSS Version 26 (IBM SPSS Statistics 2019 Armonk, NY, USA). In line with the research questions statistical analyses were based on contrasting driving performance data from the post alcohol/alcohol hangover with the no alcohol (control) condition, for both the residual and zero alcohol groups as A Priori factors, and gender and study as exploratory factors. Possible relationships between driving performance and subjective measures were also investigated. Separate MANOVAs were first performed for the driving performance and subjective data to limit type I errors, including independent variables within groups: measure (e.g., STISIM driving variable, subjective variable); alcohol (post-alcohol/hangover, no alcohol). The between groups factors were: residual alcohol (zero alcohol, residual alcohol); study (1,2,3); gender (female, male). Separate ANOVAS were run for each performance variable, and each subjective variable including alcohol and residual alcohol as factors. Significant findings with ANOVA allowed paired comparisons aligned with the research questions to be run for performance and subjective workload variables. These contrasted no alcohol (control) with the hangover condition for the zero alcohol (N = 26), and the residual alcohol (N = 26) groups. Nonparametric paired comparisons were run as distribution free confirmatory analyses. *p*-Values, two-tailed, were considered significant if *p* < 0.05. Effect size calculations for paired comparisons were undertaken using pooled variance estimates [35]. Measures of association (Pearson’s *r*) were included where P-values were at least significant at *p* < 0.01 and supported by nonparametric equivalents, in order to limit type 1 errors given the number of variables included.

## 3. Results

All N = 52 participants had zero BAC on the morning after their no alcohol consumption night. Following a night of social drinking some participants were unable to recall their exact previous night’s amount of alcohol consumption, though all participants did report they had a night of alcohol consumption. This was reflected in the results for the AHSS where group symptom means (4.7, SD 1.8) indicated they had a substantial hangover comparable with other studies. Breath analysis identified N = 26 participants who recorded 0% BAC, providing the zero alcohol group. A further N = 26 participants recorded residual alcohol with the breathalyzer, averaging 0.047% BAC (range 0.01–0.08% BAC) and made up the residual alcohol group. The AHSS hangover score was 5.1 for the zero BAC group and 4.3 for the residual alcohol group.

Multivariate analyses failed to reveal a significant effect for the exploratory factors gender and study for both the driving performance data and subjective data, and so these factors were removed whilst *A Priori* factors were retained, and data reanalyzed with the reduced model. MANOVA indicated a significant effect for alcohol for driving performance, as well as a significant effect for alcohol and an interaction between alcohol and residual alcohol for the subjective data.

### 3.1. Driving Performance

Multivariate analysis revealed an overall significant effect for alcohol (F_(1,50)_ = 19.4, *p* < 0.0001, η^2^*p* = 0.30). *A Priori* factors were included in the separate ANOVAs for the driving measures. Driving test results are presented in Table 1. Effect sizes fall within the small (0.2–0.4) to large (0.8–1+) effect size range [35].

Analysis of separate variables revealed significant differences between the alcohol hangover and no alcohol (control) conditions for components of the divided attention task including mean response time (F_(1,50)_ = 15.6, *p* < 0.0001, η^2^*p =* 0.24), incorrect responses (F_(1,50)_ = 4.85, *p* = 0.03, η^2^*p =* 0.09) and missed responses (F_(1,50)_ = 4.48, *p* = 0.04, η^2^*p =* 0.08) reflecting impairment with hangover. Driving control measures produced significant contrasts for mean speed (F_(1,50)_ = 9.00, *p* = 0.004, η^2^*p =* 0.15), center line crossing (F_(1,50)_ = 6.49, *p* = 0.01, η^2^*p =* 0.12), time off road (F(_1,50)_ = 19.9, *p* < 0.0001, η^2^*p =* 0.29) and excursions from lane (F_(1,50)_ = 14.8, *p* < 0.0001, η^2^*p =* 0.23), again reflecting poorer driving with hangover. Driving violations provided significant contrasts for accidents and collisions (F_(1,50)_ = 4.59, *p* = 0.04, η^2^*p =* 0.08), and tickets (F_(1,50)_ = 5.99, *p* = 0.02, η^2^*p =* 0.11). All these measures revealed impaired performance including slower responses, poorer steering control and more errors as well as increased traffic violations in the hangover condition compared to driving after no alcohol. Significant contrasts between the zero alcohol and residual alcohol group included mean speed (F(_1,50)_ = 4.28, *p* = 0.04, η^2^*p =* 0.08), speed limit exceedances (F_(1,50)_ = 4.59, *p* = 0.04, η^2^*p =* 0.10) and tickets (F_(1,50)_ = 4.17, *p* = 0.046, η^2^*p =* 0.08). Residual alcohol resulted in a greater mean speed, more speed limit exceedances and more tickets than the zero residual alcohol group.

A single interaction between alcohol and residual alcohol was found with accidents and collisions where the residual alcohol group experienced a greater number on hangover days compared to the zero alcohol group who showed a decrease, although with paired comparisons hangover driving failed to contrast with the no alcohol control for either the combined or zero alcohol groups, whilst the residual alcohol group had shown an increase with hangover.

For the combined groups, the results revealed significant impairments for three of the divided attention measures, indicating slower responses with more errors comprising missed responses and incorrect responses (false positives) in the hangover condition. Driving measures indicated faster driving (mean speed) with more center line crossing (going out of lane to the right), and time off road (going out of lane to the left) and more lane excursions (to left and right) in the hangover condition compared to after no alcohol. There were also a higher number of tickets (reflecting failure to stop at stop signs, jumping red lights). The paired comparisons for the zero alcohol group paralleled the combined groups except for divided attention task incorrect and missed responses, and tickets where differences were not significant. Contrasts between no alcohol and alcohol hangover for the residual alcohol group were similar to the zero alcohol group for the divided attention task, with both missed and incorrect responses failing to achieve significance, as did center line crossings in the driving control category. In contrast, there were a significantly higher number of accidents and collisions, reflecting the number of vehicles, pedestrians or animals hit, for the residual alcohol group but not for the zero alcohol group. Significant contrasts comparing hangover driving in the zero alcohol group with the residual alcohol group revealed faster driving, a greater number of speed exceedances and more tickets for the residual alcohol group.

Nonparametric paired comparisons were run as distribution free confirmatory analyses for the combined, zero and residual alcohol groups contrasting the no alcohol with the alcohol hangover condition. All statistically significant results with parametric tests were reflected in significant results with nonparametric analyses (Wilcoxon), except for missed responses for the zero alcohol group where a trend (*p* < 0.07) was observed.

### 3.2. Subjective Measures

Multivariate analysis combining the six NASA-TLX dimensions revealed an overall significant effect for alcohol (F_(1,50)_ = 54.8, *p* < 0.0001, η^2^*p* = 0.52) and alcohol x residual alcohol (F_(1,50)_ = 5.19, *p* = 0.03, η^2^*p* = 0.09) with the combined measures. Based on the literature, the focus for subjective assessment was on perceived effort and performance. These are presented in Table 1 along with the overall mean (Raw TLX) for the 6 workload dimensions. The parametric analyses provided the same significant contrasts as the nonparametric confirmatory analyses.

Analysis of the six component dimensions for the NASA-TLX with ANOVA revealed significant differences between the no alcohol (control) and alcohol hangover conditions for mental demand (F_(1,50)_ = 18.8, *p* < 0.0001, η^2^*p* = 0.27 η^2^*p*), physical demand (F_(1,50)_ = 17.5, *p* < 0.0001, η^2^*p* = 0.26), temporal demand (F_(1,50)_ = 37.8, *p* < 0.0001, η^2^*p* = 0.43), effort (F_(1,50)_ = 33.3, *p* < 0.0001, η^2^*p* = 0.40), performance (F_(1,50)_ = 8.2, *p* = 0.006, η^2^*p* = 0.14), frustration (F_(1,50)_ = 16.3, *p* < 0.0001, η^2^*p* = 0.25) and the overall means for the 6 component dimensions (F_(1,50)_ = 54.8, *p* < 0.0001, η^2^*p* = 0.52). All these measures showed an increase in perceived workload in the hangover condition compared to no alcohol. A significant alcohol x residual alcohol interaction was recorded for the overall means (F_(1,50)_ = 5.19, *p* = 0.027, η^2^*p* = 0.09), and performance (F_(1,50)_ = 4.17, *p* = 0.046, η^2^*p* = 0.08). For performance there was also a significant contrast between the zero alcohol and residual alcohol groups (F_(1,50)_ = 5.69, *p* = 0.02, η^2^*p* = 0.10) reflecting the greater increase in scores for the residual alcohol group with hangover (see Table 1.). The comparison between the zero alcohol and the residual alcohol groups indicates that the increase in performance score, reflecting poorer perceived performance, showed a significant increase with hangover in the residual alcohol group that was not seen in the zero alcohol group.

### 3.3. Associations Between Measures

The association between subjective measures and driving performance, reflecting awareness of subjective impairment, was a secondary research question. The selected subjective variables were the overall hangover rating as well as the fatigue component of the AHSS, and the NASA-TLX overall means together with the effort and performance components. The AHSS and NASA-TLX were correlated with each other as well as the driving performance measures. Variables achieving a minimum significance level of *p* < 0.01 with Pearson’s *r*, and supported by significant nonparametric equivalents for at least one group are included in Table 2. Significant results included subjective ratings of hangover symptoms (AHSS), including fatigue, as well as overall subjective workload (NASA-TLX), and both effort and performance component ratings. Effect sizes were close to or in the large effect size range (≥ 0.37) [35]. The AHSS and NASA-TLX were not associated at the required significance level (*p* < 0.01).

For residual BAC, positive correlation with subjective performance indicated higher residual alcohol levels were associated with higher performance scores which reflect poorer perceptions of performance with this component of the NASA-TLX. Higher residual BAC was also associated with greater (less negative) mean lane position scores for the residual alcohol group, possibly reflecting poorer driving by being closer to the center of the road (scored at ‘0′).

For the driving performance measures, higher scores generally indicate poorer driving. Increased mean speed and speed deviation were associated with lower hangover (AHSS, Fatigue) and workload ratings (Performance, NASA-TLX). Increased lane deviation was associated with lower performance ratings, reflecting better perceived performance, and lower subjective effort was associated with an increase in speed exceedances. However, significant associations with driving measures differed between the combined, zero and residual alcohol groups.

## 4. Discussion

This is the first study to demonstrate significant driving impairment with hangover during a typical commute to work. For the combined groups a total of 8 of the 15 driving variables including those associated with the divided attention task, as well as driving control, and driving violations which included attentional measures (e.g., tickets) showed significant impairment in the hangover condition. The means for the combined groups showed slower responses, more errors including poorer driving control and loss of attention for all the variables, even though they did not all individually achieve statistical significance. The levels of impairment during alcohol hangover seen here suggest that these are equivalent to the magnitude of driving impairment seen under conditions of alcohol intoxication at or above the 0.05–0.08% BAC level [21]. The range of neurocognitive impairments are supported by those reviewed by Kruisselbrink [19] as sensitive to alcohol hangover including perceptual motor function, complex attention and executive functioning. These results support earlier simulator studies, including the one hour highway driving test [9] and shorter motorway driving assessment [11]. The observed significant impairments indicate the need to address this issue, and also substantiate the use of our driving model for assessing hangover.

The likely explanation for the range of significant performance decrements observed on our driving test of relatively short duration is the increased cognitive demand imposed by the driving scenarios. Data from the NASA-TLX revealed that significantly increased effort was needed by the participants in both the zero and residual alcohol groups to perform (well on) the driving test, but this increased effort could not counteract driving performance impairments due to a hangover. This was also observed in the simulated highway driving study by Verster et al. [9]. Under more demanding cognitive load such as the complex driving environment used here, cognitive demand can more easily exceed capacity resulting in driving errors and decrements in performance. The impairments were therefore also recorded across a shorter driving period, rather than reflecting vigilance decrements seen in longer duration drives including the one hour driving test, in which the effects on driving were most pronounced in the second half of the test [9].

An important further aim of the study was to compare hangover driving performance for participants who had zero BAC with those who had residual levels of alcohol when driving next day in a mixed urban and rural environment during a 20 min commute to work. Although there was a residual BAC group, it is important to note that none of these participants was over the UK alcohol limit for driving (BAC 0.08%), and the mean BAC in the residual alcohol group (BAC 0.047%) was also below the 0.05% BAC limit common to several countries and recommended by the World Health Organization [1]. This observation is important as with current UK legislation all participants would be considered ‘street legal’, i.e., allowed to drive a car. When contrasting hungover driving performance of those with zero BAC and those with residual alcohol, the profiles of impairment for both these groups were similar to each other, suggesting that having residual BAC during hangover did not have a marked additional impact on driving ability. However, the residual alcohol group did drive significantly faster, resulting in significantly more speed exceedances, and they received significantly more tickets for driving violations, possibly indicating poorer attention and awareness. Increased speed, disinhibition and greater risk taking have been reported previously in studies of both alcohol intoxication, as well as alcohol hangover [3,4,11].

The impact of alcohol hangover on self awareness is important for driving and may determine whether or not a driver commutes to work next day, even though their residual alcohol BAC is within legal limits. Alcohol research has shown limited awareness of impairment in relation to driving particularly with higher BAC levels [25,26], and currently little is known in relation to alcohol hangover. Results from the present study yielded strong effects with hangover indicating that poorer driving (increased mean speed and speed deviation) was associated with reduced perceptions of hangover and fatigue (AHSS) as well as overall workload (NASA-TLX), even though workload was increased with hangover compared to no alcohol. In the hangover condition, reduced effort was associated with more speed exceedances (driving violations). Lower subjective performance ratings from the NASA-TLX, indicating perceptions of better performance, were similarly associated with poorer driving performance (increased mean speed and lane deviation). However, the finding that increased residual BAC was associated with higher ratings of poorer performance suggests some awareness, but includes the impact of the remaining alcohol. Overall, there were relatively few significant correlations between subjective measures and driving performance, this could reflect a limitation of the AHSS, which is based on individual symptoms that may not capture the complete hangover experience [36].

Taken together, these results suggest a potential lack of awareness of subjective impairment due to hangover. This was apparent in terms of lower hangover symptom scores including fatigue, and lower perceived workload including effort and better performance, being linked to more impaired driving. This is a concerning factor given that we do not currently have a direct objective measure of hangover that is equivalent to the alcohol breathalyzer and the driving performance scores show consistent impairment in the hangover condition. However, significant correlations with individual driving performance measures were limited and varied between the hangover groups, and therefore requires replication with larger samples, although the significant positive correlation with higher residual BAC associated with perceptions of poorer performance adds validity. Previous alcohol hangover studies have not shown a consistent relationship between estimated BAC for consumption the previous night, or hangover severity and objective performance on the next day [37,38]. Similarly, for this study, subjective hangover ratings (AHSS) were not significantly associated with overall subjective workload or effort and performance components.

The current study had a naturalistic design, so that participants were not monitored during the drinking session. As a result, participants consumed the type and amount of alcohol of choice, which varied between participants. Although this variability may be viewed as a limitation, it can also be regarded as a strength, because the naturalistic study design much more closely mimics a real-life drinking session, which includes non-standardized alcohol consumption and various behaviors (e.g., dancing, changing pubs) that cannot be replicated in randomized controlled trials [27]. In this context, it should be noted that the actual amount of alcohol consumed was of little relevance to the current study. More important, and irrespective of the alcohol consumed, was the premise that this would result in a next-day hangover. These criteria was met by all participants with overall hangover ratings broadly in line with earlier findings [37]. Recent research confirmed that hangovers can be experienced at any BAC level [38].

Participants were allocated to either the BAC zero or residual alcohol group after the data was collected. This was done for practical reasons, as the presence and severity of hangovers vary both within and between drinkers. Even in case of standardized alcohol administration in controlled trials, significant variability was observed in the presence and severity of hangover symptoms [38,39]. Given this, it was not possible to randomize participants and allocate them to a group before data collection took place. A related issue is how BAC was established. It has been demonstrated that breathalyzer assessments do not always correspond well with blood alcohol assessments [40]. Taking this into account, the allocation of participants to the BAC zero or residual alcohol group based on breath alcohol assessments only may not have been accurate. Future studies for which it is essential to accurately determine the presence of residual alcohol, should confirm BAC readings of zero obtained with a breathalyzer with assessments of ethanol in blood. The BAC zero group made up half of the sample, limiting the validity of the residual BAC correlations as half the 52 participants therefore had a zero score, but noting the correlations were strong with both parametric and nonparametric analyses.

Sleep disruption is a common component of alcohol hangover and naturalistic studies show reduced sleep with alcohol hangover compared to control no alcohol nights, so that overall sleep loss may be a significant component of hangover effects on waking performance next day [32]. In the current study, sleep was not recorded. Future studies should incorporate these assessments.

In the current study, participants were relatively young and inexperienced drivers. Their inclusion is warranted given that road traffic collisions are the leading cause of death for this age group [1], contributed to by a lack of observation and anticipation skills [41], increased recklessness and thrill seeking, and feelings of invincibility and over-confidence [42,43]. These combine to create ‘skill-risk optimism’ [44] whereby young drivers believe they possess high level driving skills and are unlikely to have an accident in risky-driving scenarios such as driving following a night of alcohol consumption. The research evidence, supported by the results presented here, demonstrates the opposite and may in part explain the lack of awareness of impairment amongst this population. However, what is unclear is whether the observed results in this study translate to older and more experienced drivers. Therefore, future research should also investigate the effects of alcohol hangover on driving across other age groups.

Finally, no significant sex differences were observed in the current study. This is in line with previous driving studies [9] and corresponds with the general absence in sex differences in the presence and severity of hangover symptoms at various BAC levels [45,46].

Positive aspects of the study included the sensitivity of the driving model, which included finding significant decrements in a range of driving variables including divided attention, driving control and driving violations reflecting impaired attention or risk taking, where other studies have failed to find such a wide range of effects.

## 5. Conclusions

Overall, this evaluation of driving hangover performance has been successful in demonstrating the marked impact of alcohol hangover in impairing driving performance, even though participants overall were below the legal alcohol limit for driving in several countries. The key findings are that significant impairments were seen in a range of driving measures, including complex attention and driving control for those with residual alcohol as well as those with zero alcohol, with only limited differences between the groups. The level of impairment seen here, which was comparable to driving while intoxicated at or above a BAC of 0.05%, indicates the dangers of driving whilst hungover, even when breath alcohol is at zero.

## Figures and Tables

**Table 1:** Driving Performance and Subjective Measures: Means (SDs).

	Combined Groups		Zero BAC Group		Residual Alcohol Group	
	Control	Hangover	Control	Hangover	Control	Hangover
**Divided Attention**						
Mean Response Time	2.4 (1.3)	3.5 (1.8) * [0.55]	2.3 (1.0)	3.4 (1.6) * [0.69]	2.4 (1.5)	3.7 (2.1) * [0.50]
Response Time Deviation	1.9 (1.1)	2.3 (1.0)	1.9 (1.1)	2.4 (1.2)	1.8 (1.2)	2.2 (0.8)
Correct Responses	15.2 (2.4)	14.2 (3.3)	15.5 (2.1)	14.4 (2.8)	14.9 (2.6)	14.0 (3.8)
Incorrect Responses	0.0 (0.0)	0.2 (0.5) * [0.21]	0.0 (0.0)	0.1 (0.3)	0.0 (0.0)	0.2 (0.6)
Missed Responses	1.1 (1.9)	2.1 (2.9) * [0.30]	0.9 (1.3)	2.0 (2.5)	1.3 (2.4)	2.2 (3.3)
**Driving Control**						
Mean Speed (mph)	31.7 (9.6)	35.9 (10.3) * [0.42]	29.1 (10.3)	33.7 (10.1) * [0.40]	34.3 (8.2)	38.1 (10.2) *^+^ [0.45]
Speed Deviation	10.5 (3.3)	10.6 (3.1)	10.3 (3.7)	10.5 (3.0)	10.7 (3.0)	10.7 (3.2)
Mean Lane Position	−6.4 (3.1)	−5.9 (3.2)	−7.0 (3.6)	−5.7 (3.2)	−5.8 (2.3)	−6.2 (3.2)
Lane Deviation	2.6 (1.8)	2.9 (1.7)	2.9 (2.0)	2.8 (1.6)	2.3 (1.5)	3.0 (1.8)
Center Line Crossing %	4.0 (3.1)	7.8 (10.6) * [0.36]	3.8 (2.8)	6.0 (4.7) * [0.48]	4.1 (3.4)	9.6 (14.2)
Off Road %	0.6 (0.7)	1.6 (1.7) * [0.68]	0.5 (0.6)	1.3 (1.6) * [0.61]	0.8 (0.8)	1.9 (1.8) * [0.72]
Excursions from Lane	10.6 (11.7)	18.9 (19.3) * [0.58]	9.7 (9.3)	20.2 (18.9) * [0.64]	11.5 (13.9)	17.6 (20.1) * [0.51]
**Driving Violations**						
Accidents and Collisions	1.3 (1.3)	1.8 (1.9)	1.6 (1.2)	1.1 (1.3)	0.9 (1.3)	2.5 (2.1) * [0.74]
Speed Limit Exceedances	8.7 (5.8)	9.8 (6.0)	7.0 (5.6)	9.2 (5.3)	10.4 (5.5)	10.5 (6.7)^+^
Tickets	0.8 (0.8)	1.2 (1.0) * [0.34]	0.6 (0.9)	1.0 (0.8)	1.0 (0.8)	1.4 (1.1)^+^
**Subjective Assessments**						
NASA-TLX Combined	38.4 (12.8)	53.8 (13.6) * [0.99]	40.6 (11.6)	51.3 (13.7) * [0.99]	36.2 (13.8)	56.3 (13.3) * [1.15]
Effort	41.6 (22.2)	60.5 (20.2) * [0.80]	44.4 (22.9)	60.4 (18.9) * [0.73]	38.8 (21.7)	60.6 (21.9) * [0.87]
Performance	47.4 (22.8)	60.9 (24.7) * [0.39]	47.1 (23.1)	51.0 (20.6)	47.7 (22.9)	70.8 (24.8) *^+^ [0.66]

Key: *p* < 0.05 * Control versus Hangover, + Zero BAC versus Residual Alcohol. Effect size [Cohen’s *d*] Control versus Hangover.

**Table 2:** Associations between Driving Performance and Subjective Assessments (Pearson’s *r*).

Measure	Subjective Assessment	Combined Groups	Zero BAC Group	Residual BAC Group
**Driving Control**				
Mean Speed (mph)	AHSS	−0.359 **	0.173	−0.573 **
	Fatigue	−0.460 **	−0.232	−0.561 **
	Performance	−0.247	−0.706 **	−0.104
Speed Deviation	AHSS	0.051	−0.553 **	0.317
	NASA-TLX	−0.254	−0.542 **	0.003
Mean Lane Position	Residual BAC	0.154	-	0.555 **
Lane Deviation	Performance	−0.466	−0.542 **	−0.544
**Driving Violations**				
Speed Exceedances	Effort	−0.357 **	−0.348	−0.367
**Residual BAC Assessments**				
Residual BAC	Performance	0.467 **	-	0.336

Key: ** *p* < 0.01 (minimum significance level), and nonparametric correlation also significant.
